# The role of transesophageal echocardiography in predicting technical problems and complications of transvenous lead extractions procedures

**DOI:** 10.1002/clc.23660

**Published:** 2021-07-24

**Authors:** Dorota Nowosielecka, Wojciech Jacheć, Anna Polewczyk, Łukasz Tułecki, Andrzej Kleinrok, Andrzej Kutarski

**Affiliations:** ^1^ Department of Cardiology The Pope John Paul II Province Hospital Zamość Poland; ^2^ Department of Cardiology, Faculty of Medical Sciences in Zabrze Medical University of Silesia in Katowice Zabrze Poland; ^3^ Department of Physiology, Patophysiology and Clinical Immunology Collegium Medicum of The Jan Kochanowski University Kielce Poland; ^4^ Department of Cardiac Surgery Świętokrzyskie Cardiology Center Kielce Poland; ^5^ Department of Cardiac Surgery The Pope John Paul II Province Hospital Zamość Poland; ^6^ Department of Physiotherapy, Medical College University of Information Technology and Management Rzeszów Poland; ^7^ Department of Cardiology Medical University Lublin Poland

**Keywords:** complications, effectiveness, technical difficulties, transesophageal echocardiography, transvenous leads extraction

## Abstract

**Background:**

Transesophageal echocardiography (TEE) is a useful tool in preoperative evaluation of patients undergoing transvenous lead extraction (TLE).

**Hypothesis:**

Echocardiographic phenomena may determine the difficulty and safety of the procedure.

**Methods:**

Data from 936 transesophageal examinations (TEE) performed at a high volume center in patients awaiting TLE from 2015 to 2019 were assessed.

**Results:**

TEE revealed a total of 1156 phenomena associated with the implanted leads in 697 (64.85%) patients, including: asymptomatic masses on endocardial leads (AMEL) (58.65%), vegetations (12,73%), fibrous tissue binding the lead to the vein or heart wall (33.76%), lead‐to‐lead binding sites (18.38%), excess lead loops (19.34%), intramural penetration of the lead tip (16.13%) and lead‐dependent tricuspid dysfunction (LDTD) (6.41%). Risk factors for technical difficulties during TLE in multivariate analysis were: fibrous tissue binding the lead to atrial wall (OR = 1.738; p < 0.05), to right ventricular wall (OR = 2.167; p < 0.001), lead‐to‐lead binding sites (OR = 1.628; p < 0.01) and excess lead loops (OR = 1.488; p < 0.05). Lead‐to‐lead binding sites increased probability of major complications (OR = 3.034; p < 0.05). Presence of fibrous tissue binding the lead to the superior vena cava (OR = 0.296; p < 0.05), right atrial wall (OR = 323; p < 0.05) and right ventricular wall (OR = 0.297; p < 0.05) reduced the probability of complete procedural success, whereas fibrous tissue binding the lead to the tricuspid apparatus decreased the probability of clinical success (OR = 0.307; p < 0.05).

**Conclusions:**

Careful preoperative TEE evaluation of the consequences of extended lead implant duration (enhanced fibrotic response) increases the probability of predicting the level of difficulty of TLE procedures, their efficacy and risk of major complications.

## 1 INTRODUCTION

Transvenous lead extraction (TLE) is considered first‐line strategy for the management of complications associated with cardiac implantable electronic devices (CIED).^1^, ^2^ Recently, due to the rising incidence of infectious and non‐infectious CIED‐related complications, the number of TLEs has also been increasing.^3^ According to numerous reports, the frequency of major complications of TLE ranges from 0.9% to 4.0%, and most often there is damage to the heart or venous vessels.^4^, ^5^, ^6^, ^7^ Assessment of risk factors for major complications and procedure complexity should have an impact on the selection of a suitable organizational model of the procedure and center preferment .^4^, ^5^, ^6^, ^7^ The available TLE risk stratification scales most often take into account the impact of various factors on the technical complexity of the procedure^4^, ^5^, ^6^, ^7^ or periprocedural mortality.^4^, ^5^, ^6^, ^7^ They are based on demographic and clinical data (patient age, gender, presence of co‐morbidities), type of CIED system (ICD lead, number of leads) and history of pacing (age at first implantation, number of leads designed for extraction).^4^, ^5^, ^6^, ^7^ This is the first study to assess the usefulness of new factors that may significantly affect the level of difficulty and procedure complexity as well as efficacy and complications of TLE. These factors were identified during an echocardiographic examination of patients selected for TLE due to CIED‐related complications. Echocardiography, especially transesophageal echocardiography plays a key role in the evaluation of rhythm controlling devices (PM/ICD/CRT) and remains a valuable tool for precise imaging, which is recommended by experts.^8^, ^9^, ^10^, ^11^, ^12^, ^13^, ^14^, ^15^ Although a number of studies focused on the value of preoperative TEE findings (size of vegetations, presence of asymptomatic masses on the leads), the only echocardiographic parameter discussed when estimating the procedure‐related risk was left ventricular ejection fraction (LVEF), only few studies so far have suggested that TEE can be used apart from fluoroscopy or computerized tomography^7^ to choose optimal TLE strategy,^16^, ^17^ nevertheless the effect of echocardiographic findings on procedure safety and efficacy has not been assessed.

## 2 METHODS

### 2.1 Study group

We carried out a prospective analysis of the data from 936 preoperative TEE examinations performed at a high volume center before transvenous lead extraction from June 2015 to October, 2019.

### 2.2 The extent of preoperative TEE

TEE was performed using the Philips iE33 or the GE Vivid S 70 ultrasound machine equipped with X7‐2t Live 3D or 6VT‐D probes. Images were obtained before the procedure, after general anesthesia and tracheal intubation, during preparation of the surgical field and dissection and stabilization of the leads in the device pocket. Leads were evaluated in the mid‐esophageal, inferior esophageal and modified transgastric views to visualize the right ventricle and the tricuspid valve. In order to obtain complete visualization of the structures (and assessment of lead/heart interaction) non‐standard imaging planes were sometimes required. After the procedure the results were entered into a computer database. We analyzed the number, location and course of the leads: in the superior vena cava (SVC), right atrium (RA), right ventricle (RV) (taking into account excess lead loops). We also assessed lead mobility, presence of sites at which the lead was bound to cardiac structures, lead‐to‐lead binding sites and additional masses attached to the leads. An important part of the imaging protocol was assessment of the effect of the lead on tricuspid function. Additionally, we assessed left ventricular function (LVEF), pericardial function and possible presence of structural heart disease (atrial or ventricular septal defects).

### 2.3 Definitions of echocardiographic phenomena

1. Asymptomatic masses on endocardial leads (AMEL) characterized by homogeneous echogenicity, smooth contour and varying degrees of organization. AMEL include components of connective tissue (accretions), clots, masses resembling vegetations (so‐called vegetation‐like masses). Vegetation‐like masses may be the remnant vegetations after antibiotic treatment or (less probable) organized fibrotic thrombi.^18^
2. Hyperechoic segmental thickening of the leads defined as connective tissue overgrowth (undergoing fibrosis, mineralization, crystallization and even ossification).^18^
3. Bacterial vegetations: multishaped, mobile masses of inhomogeneous echogenicity attached to the leads or/and to the neighboring anatomic structures, most frequently tricuspid leaflets. They were found only if they were accompanied by signs of a general infection. Sometimes coexisting with AMEL (vegetation‐like masses).^18^
4. Accretion–immobile fibrous connective tissue sheath around the lead causing adherence to the endocardium and vessel walls and producing images similar to segmental lead thickening but moving along with the cardiac wall.^18^
5. Excessive lead loops ‐ result of too weak fixation of the lead during implantation or lead fracture with break of insulation in the subclavian region.^18^
6. Cardiac wall perforation by the lead: visualization of the lead tip outside the heart contour, sometimes with fluid in the pericardial sac; placement of the lead tip close to the border of the pericardium is referred to as penetration.^18^

### 2.4 Transvenous lead extraction procedures

Procedures were performed in a hybrid operating room or in an operating room, using mechanical systems such as polypropylene Byrd dilator sheaths (Cook® Medical, Leechburg, PA, USA), making use of the oblique cutting edge of the tip to dissect leads from fibrous sheaths that immobilized the intravascular and/or intracardiac segment of the lead.^11^, ^19^ Procedures were performed in patients under general anesthesia with full preparation of the surgical field for cardiac surgery.

Complete procedural success, clinical success and complications of TLE were defined according to the HRS 2009 and 2017 guidelines and the 2018 EHRA expert consensus statement.^1^, ^20^, ^21^ Complete procedural success was defined as removal of all targeted leads and material, with the absence of any permanently disabling complication or procedure‐related death. Clinical success was achieved in patients with retention of a small part of the lead that did not negatively affect the outcome goals of the procedure.^1^, ^20^, ^21^

Major and minor complications were defined according to the 2018 EHRA Expert Consensus Statement on Lead Extraction.^21^

Possible technical problems during TLE include: block in lead venous entry / subclavian region, necessity utility of Evolution / TigRail (second line tool in study center), necessity of changing of venous approach for lead extraction (any reasons), impossible utility of lead venous entry approach–procedure (since beginning) using another approach, need to utilize lasso‐catheters or basket catheters, extracted lead break and broken lead remnant extraction, extracted lead break and abandonment of broken lead fragment, extracted lead fragmentation–removal in parts, lead to lead strong connection with connecting tissue scar–terrible both leads separation, collapse / fracture of Byrd dilator, dislodgement of functional lead, reeling of ICD lead coil.

### 2.5 Approval of the bioethics committee

All patients gave their informed written consent to undergo TLE and use anonymous data from their medical records, approved by the Bioethics Committee at the Regional Chamber of Physicians in Lublin no. 288/2018/KB/VII.

### 2.6 Statistical analysis

The Shapiro–Wilk test was used to test the normality of distribution of variables. For uniformity, all continuous variables are presented as the mean ± *SD*. The categorical variables are presented as number and percentage.

The significance of difference between groups were analyzed using the unpaired “*U*” Mann–Whitney test for continuous variables and the Chi^2^ test with Yates correction for categorical variables. The results of analysis were considered statistically significant at a p value <0.05. Univariate and multivariate logistic regression analysis was used to assess the impact of echocardiographic findings on TLE complexity and efficacy. Variables that in univariate analysis achieved statistical significance p < 0.05 were entered into the multivariate model. Statistical analysis was performed with Statistica version 13.0 (TIBCO Software Inc., Krakow, Poland).

## 3 RESULTS

TEE before TLE was performed in 936 patients (355 women; 37.92%), mean age 67.08 ± 14.50 years. Noninfectious indications were most common reasons for TLE (727 patients; 77.67%). Pocket infection was detected in 58 (6.20%) patients, lead‐related infective endocarditis in 151 (16.03%) patients. The study population consisted of 640 (68376%) patients with single or dual leads pacemakers (including 27 CRT‐P) and 296 (31624%) patients with ICD (including 85 CRT‐D and 165 single coil leads and 131 dual coil leads).

The remaining clinical characteristics of the patients, features of CIEDs and TLE procedures are summarized in Table 1.

**TABLE 1:** Demographic and clinical characteristics of the study population

Demographic and clinical data	All 936 patients	CIED system and history of pacing	All 936 patients
Patient age at TLE (years), mean ± *SD*	67 081 ±14.500	Presence of abandoned leads before TLE, *n* (%)	86 (9.188)
Sex (female), *n* (%)	355 (37.923)	Number of procedures before lead extraction, mean ± *SD*	1837 ±0.990
NYHA III & IV, *n* (%)	148 (15.812)	Dwell time of the oldest lead in the patient before TLE [in months], mean ± *SD*	115 843 ±77.633
LVEF >50%, *n* (%)	539 (57.585)	Cumulative dwell time of extracted leads [in years] before TLE, mean ± *SD*	17 843 ±14.530
Diabetes mellitus (any), *n* (%)	198 (21.154)	**TLE procedure complexity and difficulty**	**All 936 patients**
Renal failure (any), *n* (%)	230 (24.573)	Procedure duration (sheath to sheath) (min), mean ± *SD*	15 931 ±25.558
Charlson comorbidity index, mean ± *SD*	4886 ±3.764	Technical difficulty during TLE (any), *n* (%)	233 (24.840)
**Indications for TLE**	**All 936 patients**	Number of major technical difficulty in one patient	0.328 ±0.725
LRIE with or without pocket infection, *n* (%)	151 (16.132)	Three or more technical problems, *n* (%)	21 (2.239)
Local (pocket) infection (only), *n* (%)	58 (6.196)	**TLE procedure efficacy and outcomes**	**All 936 patients**
Non‐infectious indications, *n* (%)	727 (77.671)	Major complications (any), *n* (%)	18 (1.923)
**CIED system and history of pacing**	**All 936 patients**	Hemopericardium, *n* (%)	12 (1.282)
Number of leads in the system before TLE, mean ± *SD*	1834 ±0.639	Tricuspid valve damage during TLE, *n* (%)	6 (0.640)
Single‐, double‐chamber pacemakers, *n* (%)	640 (68376)	Rescue cardiac surgery, *n* (%)	12 (1.282)
Single, double‐chamber and cardiac resynchronization therapy ICD, *n* (%)	296 (31624)	Lack of radiological success, *n* (%)	6 (0.640)
ICD single coil leads, n (% of all ICD leads)	165 (55743)	Complete clinical success, *n* (%)	916 (97.863)
ICD double coils leads, n (% of all ICD leads)	131 (44257)	Complete procedural success, *n* (%)	917 (97.970)

*Note*: Bold paragraphs are a type of headings that are used to make the table easier to read.

Abbreviations: CIED, cardiac implantable electronic devices; LRIE, lead related infective endocarditis; LVEF, left ventricular ejection fraction; NYHA, New York Heart Association; TLE, transvenous lead extraction; ICD, implantable cardioverter‐defibrillator.

The echocardiographic findings can be divided into four basic groups:

1. Tricuspid valve dysfunction
  - Tricuspid valve dysfunction not related to the route of ventricular pacing prior to TLE (18.493%)
  - Lead‐dependent tricuspid valve dysfunction (LDTD) (6.410%)
2. Presence of any shadows on the leads (64.85% of patients)
  - Fibrous tissue binding the lead to the vena cava superior and right heart structures (33.761%): to the SVC (5.98%), to the RA wall (6.94%), to the tricuspid apparatus (9.62%) and to the RV wall (11.22%)
  - Fibrous tissue binding two leads (18.38%).
  - AMEL (46.68% of patients): fibrous tissue encasing the lead (17.094%), lead thickening (29.59%), blood clot on the lead (8.013%) vegetation‐like masses (3.953%)
  - Vegetations (12.727%)
3. Presence of excess lead loops in the heart (19.338%)
4. Perforation or penetration of the lead through the cardiac wall up to the epicardium (16.132%) (Table 2)

Comparative analysis of the impact of several echocardiographic parameters on the course and efficacy of TLE was presented in supplementary file.

**TABLE 2:** The most important preoperative echocardiographic findings in patients undergoing transvenous leads extraction

Tricuspid valve dysfunction (degree of regurgitation) ‐ excluding patients with lead dependent TV dysfunction	Average tricuspid valve regurgitation (0–4 degree), mean ± *SD*	1.454 ± 0.956
	Patients with severe tricuspid regurgitation,^3^, ^4^ *n* (%)	162 (18.493)
Lead dependent tricuspid valve dysfunction (LDTD)	Average LDTD (0–4), mean ± *SD*	3541 ± 0.594
	Patients with LDTD (any), *n* (%)	60 (6.410)
	Patients with severe LDTD (3–4) *n* (%)	58 (96.667)
Any shadows on the leads	Patients with any shadows on leads before TLE, *n* (%)	607 (64.850)
Patients with fibrous tissue binding the lead to the vena cava superior and heart structures, *n* (%)		236 (25.214)
Fibrous tissue binding the lead to the vena cava superior and heart structures	Fibrous tissue binding the lead to the heart structures (all), *n* (%)	316 (33.761)
	Fibrous tissue binding the lead to the SVC, *n* (%)	56 (5.983)
	Fibrous tissue binding the lead to the RA wall, *n* (%)	65 (6.944)
	Fibrous tissue binding the lead to the tricuspid apparatus, *n* (%)	90 (9.615)
	Fibrous tissue binding the lead to the RV wall, *n* (%)	105 (11.218)
Fibrous tissue binding two leads, *n* (%)		172 (18.377)
Patients with asymptomatic masses on endocardial leads (AMEL) (patient analysis), *n* (%)		437 (46.688)
AMEL (findings analysis)	AMEL (all), *n* (%)	549 (58.654)
	Fibrous tissue encasing the lead, *n* (% of all ILM / % of all pts)	160 (29 144 / 17.094)
	Lead thickening, *n* (% of all AMEL / % of all pts)	277 (50 455 / 29.594)
	Clot on the lead, *n* (% of all AMEL/ % of all pts)	75 (13 661 / 8.013)
	Vegetation‐like masses, *n* (% of all AMEL / % of all pts)	37 (6740 / 3.953)
Presence of vegetations	Patients with vegetations, *n* (%)	119 (12.727)
Excess lead loops in the heart	Patients with lead loops in the heart (any), *n* (%)	181 (19.338)
	Lead loops in the RA *n* (%)	138 (14.744)
	Lead loops in the TV, *n* (%)	35 (3.793)
	Lead loops in the RV or PA, *n* (%)	28 (2.991)
Perforation or penetration of the lead through the cardiac wall up to the epicardium	Perforations, *n* (%)	151 (16.132)

Abbreviations: AMEL, asymptomatic masses on endocardial leads; LDTD, lead dependent tricuspid valve dysfunction; PA, pulmonary artery; SVC, superior vena cava; RA, right atrium; RV, right ventricle; TLE, transvenous lead extraction.

### 3.1 Factors influencing the occurrence of technical difficulties–results of logistic regression analysis

Univariate regression analysis showed that the factors increasing the probability of technical difficulties were: all types of fibrous tissue binding sites: more than 2.5‐fold increase, (OR = 2.649; p < 0.001), fibrous tissue binding the lead to the SVC: 2‐fold increase (OR = 2.080; p < 0.01), to the RA: nearly 2.5‐fold increase (OR = 2.465; p < 0.001), to the TV: 2.5‐fold increase (OR = 2.533; p < 0.001), to the RV: 3‐fold increase of risk (OR = 3.080; p < 0.001), lead‐to‐lead binding sites: more than 2‐fold increase (OR = 2.263; p < 0.001), presence of AMEL: increase of 33.8% (OR = 1.338; p = 0.055) and presence of excess lead loops: increase of 76.1% (OR = 1.761; p < 0.01) (Table 3).

**TABLE 3:** Preoperative ECHO/TEE phenomena versus procedure complexity / difficulty and major complications

	Technical difficulties						Major complications					
Logistic regression	Univariate			Multivariate			Univariate			Multivariate		
	OR	95%CI	p	OR	95%CI	p	OR	95%CI	p	OR	95%CI	p
Fibrous tissue binding the lead to the heart structures (total)	2649	1.923 ‐ 3.649	<0.001				9.989	3.614 ‐ 27.61	<0.001			
Fibrous tissue binding the lead to the SVC	2.080	1.215 ‐ 3.560	<0.01	1.099	0.597 ‐ 2.021	0.761	7.972	3.086 ‐ 20.59	<0.001	2.329	0.797 ‐ 6.806	0.122
Fibrous tissue binding the lead to the RA wall	2.465	1.496 ‐ 4.063	<0.001	1.738	1.009 ‐ 2.995	<0.05	6.762	2.631 ‐ 17.38	<0.001	2.534	0.866 ‐ 7.414	0.089
Fibrous tissue binding the lead to the tricuspid apparatus	2.533	1.622 ‐ 3.957	<0.001	1.597	0.979 ‐ 2.607	0.061	6.169	2.482 ‐ 15.33	<0.001	2.570	0.878 ‐ 7.523	0.085
Fibrous tissue binding the lead to the RV wall	3.080	2.044 ‐ 4.641	<0.001	2.167	1.386 ‐ 3.390	<0.001	6.045	2.483 ‐ 14.72	<0.001	2.127	0.735 ‐ 6.160	0.163
Fibrous tissue binding two leads	2.263	1.596 ‐ 3.209	<0.001	1.628	1.102 ‐ 2.403	<0.01	7.441	3.032 ‐ 18.27	<0.001	3.034	1.094 ‐ 8.413	<0.05
AMEL	1.388	0.994 ‐ 1.803	0.055				5.009	1.670 ‐ 15.02	<0.01			
Fibrous tissue encasing the lead	1.262	0.866 ‐ 1.839	0.226				2.971	1.210 −7.30	<0.05	2.150	0.808 ‐ 5.723	0.125
Lead thickening	1.582	1.155 ‐ 2.167	<0.01	1.208	0.861 −1.696	0.274	4.958	1.977 −12.44	<0.001	2.253	0.821 ‐ 6.185	0.114
Vegetations	0.705	0.431 ‐ 1.152	0.162				0.761	0.175 −3.322	0.717			
LDTD	0.989	0.513 ‐ 1.907	0.973				0.713	0.094 ‐ 5.413	0.743			
Lead loops in the heart (any)	1.761	1.242 ‐ 2.497	<0.001	1.488	1.031 ‐ 2.147	<0.05	1.267	0.458 ‐ 3.510	0.648			

Abbreviations: AMEL, asymptomatic masses on endocardial leads; LDTD, lead dependent tricuspid valve dysfunction; PA, pulmonary artery; SVC, superior vena cava; RA, right atrium; RV, right ventricle; TLE, transvenous lead extraction.

Multivariate analysis showed that fibrous tissue binding the lead to the RA (OR = 1.738; p < 0.05), to the RV (OR = 2.167; p < 0.001), lead‐to‐lead binding sites (OR = 1.628; p < 0.01) and excess lead loops (OR = 1.488; p < 0.05) were the strongest predictors of technical difficulties. Fibrous tissue binding the lead to the TV approached the borderline of significance (OR = 1.597; p = 0.061) (Table 3).

### 3.2 Factors influencing the occurrence of major complications – results of logistic regression analysis

Univariate logistic regression analysis showed that the factors increasing the probability of major complications were: all types of fibrous tissue binding sites: 10‐fold increase (OR = 9.989; p < 0.001), fibrous tissue binding the lead to the SVC: 8‐fold increase (OR = 7.972; p < 0.001), to the RA: nearly 7‐fold increase (OR = 6.762; p < 0.001), to the TV: 6‐fold increase (OR = 6.169; p < 0.001), to the RV: 6‐fold increase (OR = 6.045; p < 0.001), lead‐to‐lead binding sites: 7.5‐fold increase (OR = 7.441; p < 0.001) and the presence of AMEL: 5‐fold increase (OR = 5.009; p < 0.01), and especially fibrous tissue encasing the lead: 3‐fold increase (OR = 2.971; p < 0.05) and lead thickening: 5‐fold increase (OR = 4.958; p < 0.001).

Multivariate analysis showed that lead‐to‐lead binding sites were the strongest predictor of major complications (3‐fold increase; OR = 3.034; p < 0.05). Fibrous tissue binding the lead to the RA: 2.5‐fold increase (OR = 2.534; p = 0.089) and to the TV: 2.5‐fold increase (OR = 2.570; p = 0.085) approached the borderline of significance (Table 3).

### 3.3 Factors affecting complete clinical success–results of logistic regression analysis

In univariate logistic regression analysis the factors that reduced the probability of clinical success were as follows: all types of fibrous tissue binding sites: decrease of 88.7% (OR = 0.113; p < 0.001), fibrous tissue binding the lead to the SVC: decrease of 85.6% (OR = 0.144; p < 0.001), to the RA: decrease of 83.1% (OR = 0.169; p < 0.001), to the TV: decrease of 80.7% (OR = 0.153; p < 0.001), to the RV: decrease of 80.7% (OR = 0.193; p < 0.001), lead‐to‐lead binding sites: decrease of 83.2% (OR = 0.168; p < 0.001) and the presence of AMEL: decrease of 76.4% (OR = 0.236; p < 0.01), including the presence of fibrous tissue encasing the lead: decrease of 63.7% (OR = 0.363; p < 0.05) and lead thickening: decrease of 81.2% (OR = 0.188; p < 0.001).

Multivariate analysis showed that fibrous tissue binding the lead to the TV (OR = 0.307; p < 0.05) was the strongest predictor of clinical success. Lead‐to‐lead binding sites (OR = 0.378; p = 0.054) and lead thickening (OR = 0.385; p = 0.059) approached the borderline of significance (Table 4).

**TABLE 4:** TLE efficacy and most important preoperative TEE findings

	Complete procedural success					Complete clinical success						
Logistic regression	Univariate			Multivariate			Univariate			Multivariate		
	OR	95%CI	p	OR	95%CI	p	OR	95%CI	p	OR	95%CI	p
Fibrous tissue binding the lead to the heart structures (total)	0.080	0.026–0.242	<0.001				0.113	0.044–0.290	<0.001			
Fibrous tissue binding the lead to the SVC	0.149	0.055–0.403	<0.001	0.296	0.092–0.958	<0.05	0.144	0.057–0.365	<0.001	0.448	0.155–1.301	0.139
Fibrous tissue binding the lead to the RA wall	0.175	0.065–0.472	<0.001	0.323	0.107–0.971	<0.05	0.169	0.067–0.427	<0.001	0.428	0.147–1.245	0.119
Fibrous tissue binding the lead to the tricuspid apparatus	0.188	0.073–0.483	<0.001	0.474	0.156–1.438	0.187	0.153	0.064–0.366	<0.001	0.307	0.109–0.864	0.025
Fibrous tissue binding the lead to the RV wall	0.151	0.061–0.375	<0.001	0.297	0.102–0.862	<0.05	0.193	0.082–0.459	<0.001	0.545	0.190–1.564	0.259
Fibrous tissue binding two leads	0.275	0.112–0.674	<0.01	0.749	0.259–2.170	0.594	0.168	0.073–0.391	<0.001	0.378	0.140–1.020	0.054
AMEL	0.577	0.233–1.427	0.223				0.188	0.063–0.561	<0.01			
Fibrous tissue encasing the lead	0.853	0.281–2.589	0.779				0.363	0.150–0.881	<0.05	0.500	0.190–1.319	0.161
Lead thickening	0.505	0.207–1.235	0.134				0.188	0.076–0.466	<0.001	0.385	0.143–1.039	0.059
Vegetations	1.243	0.283–5.450	0.773				1.455	0.336–6.296	0.615			
LDTD	0.265	0.086–0.821	<0.05	0.316	0.092–1.086	0.067	1.547	0.204–11.717	0.672			
Lead loops in the heart (any)	0.451	0.177–1.148	0.095				0.890	0.327–2.421	0.819			

Abbreviations: AMEL, asymptomatic masses on endocardial leads; LDTD, lead dependent tricuspid valve dysfunction; PA, pulmonary artery; SVC, superior vena cava; RA, right atrium; RV, right ventricle; TLE, transvenous lead extraction.

### 3.4 Factors affecting complete procedural success – results of logistic regression analysis

Univariate logistic regression analysis showed that the factors that reduced the probability of clinical success were as follows: all types of fibrous tissue binding sites: decrease of 92% (OR = 0.080; p < 0.001), fibrous tissue binding the lead to the SVC: decrease of 85.1% (OR = 0.149; p < 0.001), to the RA: decrease of 82.5% (OR = 0.175; p < 0.01), to the TV: decrease of 81.2% (OR = 0.188; p < 0.01), to the RV: decrease of 84.9% (OR = 0.151; p < 0.001), lead‐to‐lead binding sites: decrease of 72.5% (OR = 0.275; p < 0.01) and the presence of LDTD: decrease of 73.5% (OR = 0.265; p < 0.05).

Multivariate analysis showed that fibrous tissue binding the lead to the SVC (OR = 0.296; p < 0.05), to the RA (OR = 0.323; p < 0.05), to the RV (OR = 0.297; p < 0.05) were the strongest predictors of procedural success. LDTD approached the borderline of significance (OR = 0.316; p = 0.067) (Table 4).

## 4 DISCUSSION

Transesophageal echocardiography is a very important part of patient evaluation before transvenous lead extraction because of its ability to detect phenomena related to the presence of leads in the heart and potentially affecting the course, safety and efficacy of the procedure.^1^, ^2^ In this study the echocardiographic findings in patients with CIEDs were divided into four basic groups: 1.tricuspid valve dysfunction 2. presence of any shadows on the leads before TLE 3. presence of excess lead loops in the heart and 4. perforation or penetration of the lead through the cardiac wall up to the epicardium. From the viewpoint of the planned transvenous lead extraction, the most important findings were the many faces of fibrosis associated with the leads varying in location and intensity. The degree of resistance/hardness and fibrosis ‐ the most common enemy of the operator ‐ appeared to determine the level of difficulty and safety of the procedure. There is a large volume of published studies describing the presence of mobile masses attached to the leads visualized by TTE, TEE and ICE in asymptomatic patients.^22^, ^23^ In this study additional masses on endocardial leads (AMEL) were defined as fibrous connective tissue (accretions), clots, vegetations‐like massess. Similar to Golzio PG et al.,^23^ we also took into consideration lead thickening and hyperechogenicity, frequently present (29.594%) in patients undergoing TLE (Figure S1 supplementary file).

Visualization of excess lead loops was another component of the TEE evaluation of patients before TLE. Lead looping was usually a result of long‐term contact with the myocardium, and hence a stronger adhesion involving longer segments. In this study excess lead loops were most common in the RA (138; 14.744% cases), and least frequent in the RV and the TPA (35; 3.793% cases). The presence of excess lead loops did not affect the procedure‐related risk, although it increased the level of complexity.

Lead loops are very well visible on fluoroscopy, but the advantage of TEE is that it permits detection of fibrous tissue binding the lead loops to the heart walls and its possible impact on the tricuspid apparatus (Figure S2 supplementary file).

The presence of excess lead loops in the heart is on the one side a result of suboptimal lead positioning (no last look after the leads become lodged in the tissue), too weak tightening of the sutures, no radiological verification of lead positioning until device replacement when the mere pulling back is already impossible.

**FIGURE 1:**
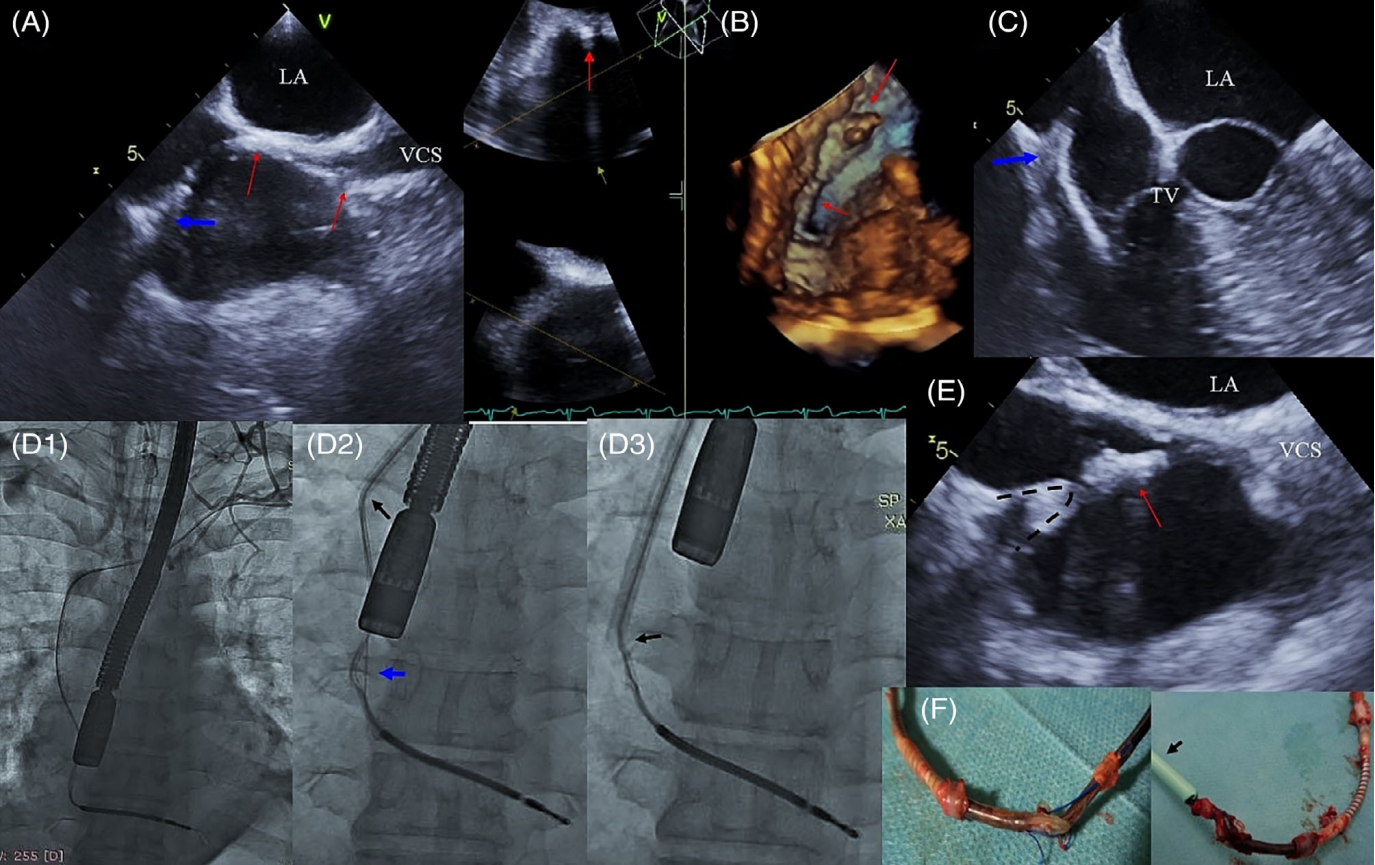
Build‐up around an ICD lead visualized on TEE and fluoroscopy and its consequences during TLE. (A) TEE (2D, ME‐bicaval) Segmental thickening of the ICD lead with three binding sites in the RA wall (arrows), additionally a blue arrow points to the binding site and conductor externalization. (B) TEE (2D and 3D, ME ‐ bicaval) The thickened ICD lead attached to the IAS (arrows). (C) TEE (2D, ME RV ‐ Inflow) The ICD lead, hyperechoic, thickened, over the TV bound to the lateral atrial wall at the site of externalization (blue arrow). (D) D1‐Evaluation of ICD lead position and venous patency before TLE. D1–Imaging during TLE–well visible site of externalization (blue arrow), the tip of Byrd's dilator marked with a black arrow. D3–significant pulling on the ICD lead during TLE. (E) TEE (2D, bicaval) The moment of pulling on the thickened ICD lead (red arrow) seen on fluoroscopy. D3–significant pulling on the RA wall and the separation of pericardial layers. (F) The extracted lead with multiple fragments of the connective tissue, the site of externalization and a dilator (black arrow)

Another echocardiographic finding that is, fibrous tissue binding the lead to the adjacent heart and vessel structures deserves discussion, as so far the problem has received scant attention in the research literature .^20^ In this study, fibrous tissue binding sites were recognized on inspection of the lead location and mobility with respect to one another and cardiac structures, looking for such signs as immediate vicinity, thickening and lead/heart wall mobility during cardiac work (Figure 1).

The connective tissue on the leads (scar tissue build‐up around the lead, fibrous tissue binding sites, accretions) is visible on TEE as lead thickening resulting in the formation of sites at which the leads are bound to one another after being in direct contact over an extended period of time. The imaging of this phenomenon has important implications for the course of the TLE procedure. During the extraction procedure the direct pulling on the wall at the binding site may be too strong and cause inadvertent pulling on and uncontrolled removal of the other lead, risking a tear of the heart wall with cardiac tamponade or hemopericardium as the end result.

The originality of this study is that it explores the impact of TEE assessment before TLE on the course of the procedure. Multivariate analysis showed that lead‐to‐lead binding sites were the strongest predictive factor which caused a 3‐fold increase in the probability of major complications during TLE. The presence of fibrous tissue binding the lead to the atrial wall and tricuspid valve approached the borderline of significance. The presence of binding sites in the RV wall caused a nearly 2‐fold increase in the risk of technical difficulties, thus increasing the degree of procedure complexity. The probability of technical difficulty increased also in the presence of excess lead loops, fibrous tissue binding the lead to the RA wall and lead‐to‐lead binding sites. The presence of binding sites in the tricuspid apparatus and lead‐to‐lead adhesion on the borderline of statistical significance reduced the chances of complete clinical success. The chances of procedural success were also reduced in relation with the presence of binding sites in the SVC, RA and lead‐to‐lead adhesions, whereas lead‐dependent tricuspid dysfunction approached the borderline of significance.

There are numerous studies^4^, ^5^, ^6^, ^7^ which on the basis of demographic data (age, sex), clinical information (indications, accompanying diseases, heart sufficiency), information about PM/ICD/CRT devices (number and type of leads) and history of pacemaker therapy (age of leads and route of implantation) show that initial patient assessment may identify the individuals in whom TLE may be more difficult or associated with the occurrence of major complications. Only few studies using scoring systems provide a more precise prediction of the level of procedure difficulty or estimate the true risk.^6^, ^7^ A review of the literature shows that so far echocardiographic findings have not been analyzed with respect to prediction of technical difficulties associated with TLE and complications of the procedure. Only one paper demonstrated that low LVEF was a predictor of major complications,^6^ another paper documented an eventful postoperative course in patients with right ventricular dysfunction.^4^ The evidence from another study suggests that information from CT examination may be useful for estimating procedure difficulty.^8^ Yet another study implies that accurate Doppler blood flow measurements in the SVC may identify patients with significant lead fibrosis requiring powered sheaths for successful removal. Although numerous papers have emphasized the role of the connective tissue (scar tissue binding the lead to the SVC and heart wall) in estimating procedure complexity and its complications,^3^, ^6^, ^7^ to the best of our knowledge we are the first to use the information about the degree of connective tissue build‐up to predict technical difficulties and risk of major complications associated with TLE.

When developing a risk calculator for prediction of complications (SAFeTy TLE)^7^ we found out that lead‐to‐lead binding site was an extremely important prognostic factor, however other information (S, sum of lead dwell times; A, anemia, Fe, female; T, treatment [previous procedures], Y , young patients) appeared more significant in multivariate analysis. We are of the opinion that all forms of connective tissue response (scar tissue binding the lead to the vein and heart structures, lead‐to‐lead adhesion) are extremely significant factors that increase procedure complexity and its radiological efficacy, however they do not necessarily translate into major complications at an experienced high volume center. Nevertheless, TEE before TLE should become a tool that provides additional information about procedure‐related risk. Specific findings from echocardiography could have an impact on the procedure in term of logistic or in term of procedural steps.

## 5 LIMITATIONS

This is a single‐center, observational, prospective study. TLE was performed using mechanical systems without laser energy. Comparison of diagnostic sensitivity of TEE and ICE was not the aim of the study.

## 6 CONCLUSIONS

Enhanced inflammatory response in the form of connective tissue sheaths that surround the leads and their cardiac and vascular binding sites very significantly affect the degree of TLE complexity, its efficacy defined as clinical, procedural and radiological success and the occurrence of major complications. Careful preoperative TEE evaluation of the consequences of extended lead implant duration (enhanced fibrotic response) by means of preoperative transesophageal echocardiography increases the probability of predicting the level of TLE complexity, its efficacy defined by clinical, procedural and radiological success and the risk of major complications associated with TLE.

## CONFLICT OF INTEREST

The authors declare no potential conflict of interest.

## Supporting information

**AppendixS1**: Supporting information

## Data Availability

Research data are not shared
