# Supplementary material for: The role of transesophageal echocardiography in predicting technical problems and complications of transvenous lead extractions procedures
Source: Clin Cardiol. 2021 Jul 24;44(9):1233–42. doi: 10.1002/clc.23660 (PMC8427997; doi:10.1002/clc.23660)
Supplement: Supplementary file 1 — AppendixS1: Supporting information [file CLC-44-1233-s001.docx]

**The role of transesophageal echocardiography in predicting technical problems and complications of transvenous lead extractions procedures**

Authors: Dorota Nowosielecka ^1^, Wojciech Jacheć ^2^ , Anna Polewczyk ^3,4^ , Łukasz Tułecki ^5^, Andrzej Kleinrok ^1,6^ , Andrzej Kutarski ^7^

^1^ Department of Cardiology The Pope John Paul II Province Hospital, Zamość, Poland

^2^ Medical University of Silesia in Katowice, Faculty of Medical Sciences in Zabrze, 2^nd^Department of Cardiology, Zabrze, Poland

^3^ Collegium Medicum of The Jan Kochanowski University, Kielce, Poland

^4^ Department of Cardiac Surgery, Świętokrzyskie Cardiology Center, Kielce, Poland

^5^ Department of Cardiac Surgery The Pope John Paul II Province Hospital, Zamość, Poland

^6^ University of Information Technology and Management, Rzeszów, Poland

^7^ Department of Cardiology, Medical University, Lublin, Poland

Comparative analysis of the impact of several echocardiographic parameters on the course and efficacy of TLE showed that higher LVEF (>50%) was associated with increased duration of the procedure, more common technical difficulties and major complications related to cardiac and vascular injury (hemopericardium, rescue cardiac surgery), which resulted in lower rates of complete clinical success in this group of patients (96.660% vs. 99.496% in patients with LVEF<50%; p= 0.006).

Analysis of the course of TLE in patients with tricuspid regurgitation (TR) showed lower rates of complete procedural success in the presence of severe TR as compared with mild TR (95.455% vs. 98.743%; p=0.006).

The presence of any shadows on the leads was associated with increased duration of the procedure, more common technical difficulties and major complications (hemopericardium) together with lower rates of complete procedural success (97.035% vs. 99.696%, p=0.012).

The presence of AMEL was associated with increased duration of the procedure, technical difficulties (p=0.055), more frequent major complications (hemopericardium, rescue cardiac surgery) and lower rates of complete clinical success (96.110% vs. 99.398%; p= 0.001).

The presence of strong connective tissue scar binding the lead to heart structures was associated with increased duration of the procedure, technical difficulties, more major complications (associated with heart and vessel injury, and tricuspid valve injury during TLE), more frequent lack of radiological success (2.542% vs. 0.000%; p<0.001) and lower rates of complete procedural success (93.644% vs. 99.286%; p<0.001) and complete clinical success (93.644% vs. 99.286%; p<0.001).

The discussed results are presented in the table.

**Table TEE abnormalities (important selected findings) and TLE difficulty and effectiveness**

| **TLE procedure difficulty and complexity** | All patients | According to LVEF (below and above 50%) | According to severe tricuspid valve regurgitation presence | According to any shadows on leads presence | According to asymptomatic masses on endocardial leads – AMEL presence | According to  fibrous tissue binding the lead to the heart structures presence (any) |
| --- | --- | --- | --- | --- | --- | --- |
| **Selected subgroups of patients for comparison** |  | Below (n= 399)  vs  above (n=539) | 0-2+ (n=718)  vs  3-4+ (n=220) | No (n=286)  vs  Yes (n=552) | No (n=695)  vs  Yes (n=243) | No (n=701)  vs  Yes (n=237) |
| Procedure duration (sheath to sheath) (min) mean ± SD | 15,931  ±25,558 N=938 | 13,178  ±20,973  N=399  vs  17,968  ±28,328  N=539  **P<0,001** | 15,223  ±24,228  N=718  vs  18,241  ±29,428  N=220  P=0,153 | 12,940  ±20,069  N=386  vs  18,022  ±28,614  N=552  **P<0,001** | 15,233  ±21,265 N=695  vs  17,926  ±35,028 N=243  P=0,275 | 12,164  ±17,386  N=701  vs  27,072  ±39,116  N=237  **P<0,001** |
| Technical problem during TLE (any) n (%) | 233  (24,840) N=938 | 79  (19,799)  N=399  vs  154  (28,571)  N=539  **P=0,003** | 184  (25,627)  N=718  vs  49  (22,273)  N=220  P=0,356 | 80  (20,725)  N=386  vs  153  (27,717)  N=552  **P=0,018** | 171  (24,604)  N=695  vs  62  (25,514)  N=243  P=0,844 | 139  (19,829)  N=701  vs  94  (39,662)  N=237  **P<0,001** |
| Number of big technical problems in one patient | 0,328  ±0,725 N=938 | 0,233  ±0,604  N=399  vs  0,399  ±0,796  N=539  **P<0,001** | 0,320  ±0,681  N=718  vs  0,355  ±0,856  N=220  P=0,716 | 0,259  ±0,624  N=386  vs  0,377  ±0,785  N=552  P=0,103 | 0,321  ±0,697  N=695  vs  0,350  ±0,801  N=243  P=0,828 | 0,225  ±0,564  N=701  vs  0,633  ±1,011  N=237  **P<0,001** |
| Three or more technical problems n (%) | 21  (2,239) N=938 | 0,233  ±0,604  N=399  vs  0,399  ±0,796  N=539  **P<0,001** | 14  (1,950)  N=718  vs  7  (3,182)  N=220  P=0,412 | 7  (1,813)  N=386  vs  14  (2,536)  N=552  P=0,609 | 15  (2,158)  N=695  vs  6  (2,469)  N=243  P=0,976 | 9  (1,284)  N=701  vs  12  (5,063)  N=237  **P=0,002** |
| **TLE efficacy and complications** | all | According to LVEF (below and above 50%) | According to severe tricuspid valve regurgitation presence | According to any shadows on leads presence | According to asymptomatic masses on endocardial leads – AMEL presence | According to  fibrous tissue binding the lead to the heart structures presence (any) |
| **Selected subgroups of patients for comparison** |  | Below (n= 399)  vs  above (n=539) | 0-2+ (n=718)  vs  3-4+ (n=220) | No (n=286)  vs  Yes (n=552) | No (n=695)  vs  Yes (n=243) | No (n=701)  vs  Yes (n=237) |
| Major complications (any) n (%) | 21  (2,239) N=938 | 3  (0,752)  N=399  vs  18  (3,340)  N=539  **P=0,015** | 18  (2,507)  N=718  vs  3  (1,364)  N=220  P=0,458 | 3  (0,777)  N=386  vs  18  (3,261)  N=552  **P=0,021** | 12  (1,727)  N=695  vs  9  (3,704)  N=243  P=0,123 | 5  (0,713)  N=701  vs  16  (6,751)  N=237  **P<0,001** |
| Hemopericardium n (%) | 13  (1,386) N=938 | 0  (0,000)  N=399  vs  13  (2,412)  N=539  **P=0,005** | 11  (1,532)  N=718  vs  2  (0,909)  N=220  P=0,717 | 1  (0,259)  N=386  vs  12  (2,174)  N=552  **P=0,029** | 7  (1,007)  N=695  vs  6  (2,469)  N=243  P=0,174 | 4  (0,571)  N=701  vs  9  (3,797)  N=237  **P=0,008** |
| Tricuspid valve damage during TLE n (%) | 6  (0,640) N=9 | 1  (0,251)  N=399  vs  5  (0,928)  N=539  P=0,383 | 6  (0,836)  N=718  vs  0  (0,000)  N=220  P=0,381 | 1  (0,259)  N=386  vs  5  (0,906)  N=552  P=0,420 | 4  (0,576)  N=695  vs  2  (0,823)  N=243  P=0,960 | 0  (0,000)  N=701  vs  6  (2,532)  N=237 |
| Rescue cardiac surgery  n (%) | 16  (1,706) N=938 | 2  (0,501)  N=399  vs  14  (2,597)  N=539  **P=0,028** | 13  (1,811)  N=718  vs  3  (1,364)  N=220  P=0,881 | 2  (0,518)  N=386  vs  14  (2,536)  N=552  P=0,036 | 8  (1,151)  N=695  vs  8  (3,292)  N=243  P=0,054 | 5  (0,713)  N=701  vs  11  (4,641)  N=237  **P<0,001** |
| Complete radiological success n (%) | 919  (97,974) N=938 | 394  (98,747)  N=399  vs  525  (97,403)  N=539  P=0,226 | 709  (98,747)  N=718  vs  210  (95,455)  N=220  **P=0,006** | 382  (98,964)  N=386  vs  537  (97,283)  N=552  **P=0,026** | 680  (97,842)  N=695  vs  239  (98,354)  N=243  P=0,823 | 698  (99,572)  N=701  vs  221  (93,249)  N=237  **P<0,001** |
| Partial radiological success (remained tip or < 4 cm lead fragment) n (%) | 13  (1,386) N=938 | 3  (0,752)  N=399  vs  10  (1,855)  N=539  P=0,252 | 4  (0,557)  N=718  vs  9  (4,091)  N=220  **P=0,003** | 3  (0,777)  N=386  vs  10  (1,812)  N=552  P=0,294 | 10  (1,439)  N=695  vs  3  (1,235)  N=243  P=0,933 | 3  (0,428)  N=701  vs  10  (4,219)  N=237  **P<0,001** |
| Lack of radiological success  n (%) | 6  (0,640) N=938 | 2  (0,501)  N=399  vs  4  (0,742)  N=539  P=0,966 | 5  (0,696)  N=718  vs  1  (0,455)  N=220  P=0,928 | 1  0,259  N=386  vs  5  (0,906)  N=552  P=0,420 | 5  (0,719)  N=695  vs  1  (0,412)  N=243  P=0,960 | 0  (0,000)  N=701  vs  6  (2,532)  N=237 |
| Complete clinical success n (%) | 915  (97,548) N=938 | 395  (98,997)  N=399  vs  520  (96,475)  N=539  **P=0,024** | 699  (97,354)  N=718  vs  216  (98,182)  N=220  **P=0,023** | 382  (98,964)  N=386  vs  533  (96,558)  N=552  P=0,033 | 681  (97,986)  N=695  vs  234  (96,296)  N=243  P=0,221 | 695  (99,144)  N=701  vs  220  (92,827)  N=237  **P<0,001** |
| Complete procedural success  n (%) | 918  (97,868) N=938 | 394  (98,747)  N=399  vs  524  (97,217)  N=539  P=0,169 | 707  (98,468)  N=718  vs  211  (95,909)  N=220  **P=0,042** | 382  (98,964)  N=386  vs  536  (97,101)  N=552  P=0,087 | 679  (97,698)  N=695  vs  239  (98,354) N=243  P=0,925 | 697  (99,429)  N=701  vs  221  (93,249)  N=237  **P<0,001** |

Abbreviations : AMEL- asymptomatic masses on endocardial leads, LVEF- left ventricular ejection fraction TEE- transesophageal echocardiography, TLE- - transvenous lead extraction

**Figure S1**

Additional masses associated with endocardial leads detected on preprocedural TEE


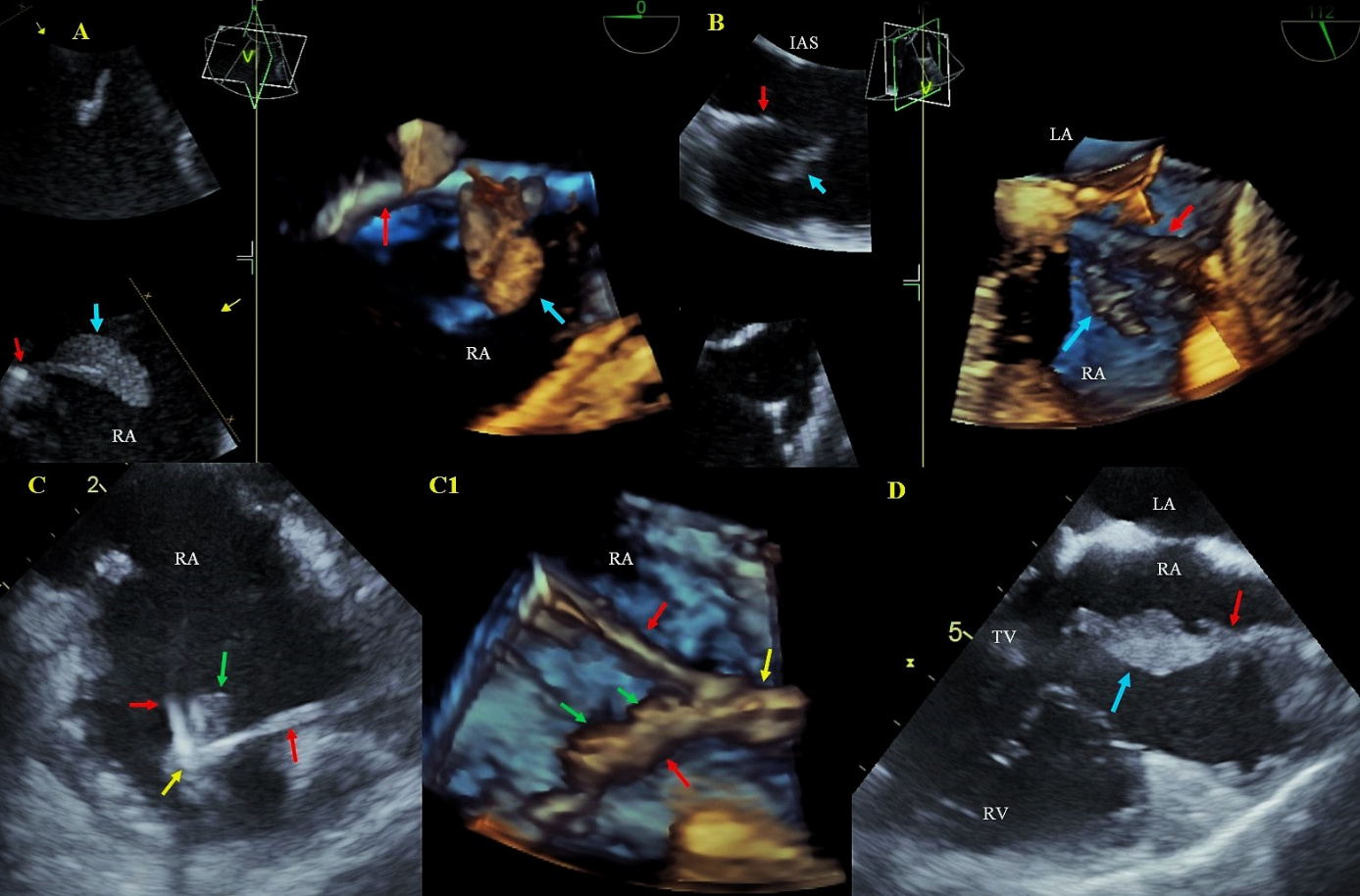


1. TEE (2D and 3D, ME- bicaval) In the RA an additional mass (red arrow) attached to the lead, a mobile mass (blue arrows) representing a bacterial vegetation
2. TEE (2D and 3 D, ME- bicaval) Segmental lead thickening (red arrow) in the atrial course with an additional mobile mass (blue arrow) representing the connective tissue build-up (accretion, scar)
3. TEE (2D, ME- modified) In the RA cavity close to the SVC orifice an echo of two leads (red arrows) with additional irregular masses (green arrows) at lead-to-lead binding site (yellow arrow) representing clots. C1 – 3D imaging
4. TEE (2D, ME- modified to visualize right cardiac chambers) In the RA a mass attached to the lead (red arrow) that may represent a pseudo vegetation (blue arrow)

**Figure S2**

Consequences of excess ventricular lead loops on TEE examination


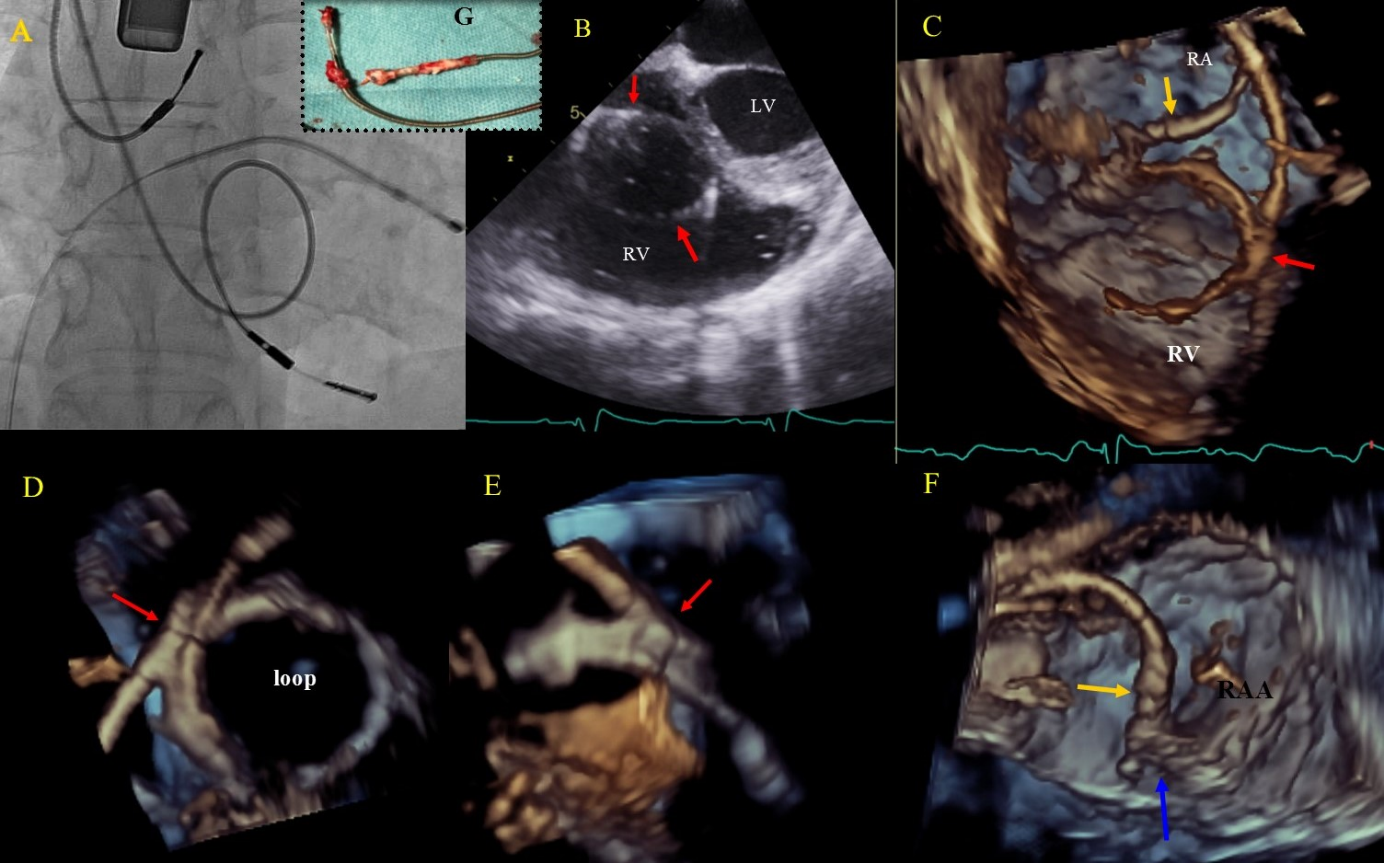


1. Fluoroscopy. Ventricular lead loop in the RV cavity not affecting TV function
2. TEE (2D ME RV Inflow-Outflow modified) Ventricular lead loop forming a closed circle, with segmental thickening
3. TEE (3D modified) The atrial lead (yellow arrow) directed towards the RAA with a well visible distal segment and a loop formed by the ventricular lead in the RV with lead-to-lead binding site in the distal segment (red arrow)
4. TEE (3D) Zoom in on the distal segment within the loop confirming lead-to-lead binding site (red arrow)
5. The same as in Figure 2D
6. TEE (3D ME – bicaval) The atrial lead (yellow arrow) implanted in the RAA wall with a visible binding site in the distal segment (blue arrow)
7. Extracted leads surrounded by the connective tissue sheath
